# Medication Use before, during, and after Pregnancy among Women with Eating Disorders: A Study from the Norwegian Mother and Child Cohort Study

**DOI:** 10.1371/journal.pone.0133045

**Published:** 2015-07-22

**Authors:** Angela Lupattelli, Olav Spigset, Leila Torgersen, Stephanie Zerwas, Marianne Hatle, Ted Reichborn-Kjennerud, Cynthia M. Bulik, Hedvig Nordeng

**Affiliations:** 1 PharmacoEpidemiology and Drug Safety Research Group, School of Pharmacy, PharmaTox Strategic Initiative, Faculty of Mathematics and Natural Sciences, University of Oslo, Oslo, Norway; 2 Department of Clinical Pharmacology, St Olav’s University Hospital, Trondheim, Norway; 3 Department of Laboratory Medicine, Children’s and Women’s Health, Norwegian University of Science and Technology, Trondheim, Norway; 4 Division of Mental Health, Norwegian Institute of Public Health, Oslo, Norway; 5 UNC Center of Excellence for Eating Disorders, Department of Psychiatry, University of North Carolina at Chapel Hill, Chapel Hill, North Carolina, United States of America; 6 Clinic for Eating Disorders, Oslo, Norway; 7 Department of Psychiatry, University of Oslo, Oslo, Norway; 8 Department of Nutrition, University of North Carolina at Chapel Hill, Chapel Hill, North Carolina, United States of America; 9 Department of Medical Epidemiology and Biostatistics, Karolinska Institutet, Stockholm, Sweden; Medical University of Vienna, AUSTRIA

## Abstract

**Introduction:**

Little is known about medication use among women with eating disorders in relation to pregnancy.

**Aims:**

To explore patterns of and associations between use of psychotropic, gastrointestinal and analgesic medications and eating disorders in the period before, during and after pregnancy.

**Method:**

This study is based on the Norwegian Mother and Child Cohort Study (MoBa). A total of 62,019 women, enrolled at approximately 17 weeks' gestation, had valid data from the Norwegian Medical Birth Registry and completed three MoBa questionnaires. The questionnaires provided diagnostic information on broadly defined anorexia nervosa (AN), bulimia nervosa (BN), binge eating disorder (BED) and recurrent self-induced purging in the absence of binge eating (EDNOS-P), along with self-reported use of medication six months before, during, and 0–6 months after pregnancy.

**Results:**

The prevalence of eating disorder subtypes before and/or during pregnancy was: 0.09% AN (n = 54), 0.94% BN (n = 585), 0.10% EDNOS-P (n = 61) and 5.00% BED (n = 3104). The highest over-time prevalence of psychotropic use was within the AN (3.7–22.2%) and EDNOS-P (3.3–9.8%) groups. Compared to controls, BN was directly associated with incident use of psychotropics in pregnancy (adjusted RR: 2.25, 99% CI: 1.17–4.32). Having AN (adjusted RR: 5.11, 99% CI: 1.53–17.01) or EDNOS-P (adjusted RR: 6.77, 99% CI: 1.41–32.53) was directly associated with use of anxiolytics/sedatives postpartum. The estimates of use of analgesics (BED) and laxatives (all eating disorders subtypes) were high at all time periods investigated.

**Conclusions:**

Use of psychotropic, gastrointestinal, and analgesic medications is extensive among women with eating disorders in the period around pregnancy. Female patients with eating disorders should receive evidence-based counseling about the risk of medication exposure versus the risk of untreated psychiatric illness during pregnancy and postpartum.

## Introduction

Eating disorders are serious mental illnesses primarily affecting women of childbearing age. It is estimated that 0.9%, 1.5%, and 3.5% of the female population experience anorexia nervosa (AN), bulimia nervosa (BN), or binge eating disorder (BED), respectively, over the life time [1]. An active or past eating disorder does not preclude a woman from getting pregnant. Even women with AN, despite the high prevalence of menstrual disturbances (up to 90%), may become pregnant during an intermittent phase of regular ovulation, or during the first ovulation after a period of amenorrhea [2]. The fertility rate and parity among women with eating disorders is comparable to that observed in the general population, although women with BN seem to undergo fertility treatments more frequently than healthy controls [3–5]. On the other hand, pregnancy is often unplanned among women suffering from AN [6].

During pregnancy, up to 7.5% of women may meet the diagnostic criteria for an eating disorder [7]. Eating disorders can negatively affect pregnancy outcome and not least maternal health during pregnancy and postpartum. Indeed, women presenting eating disorder symptoms during pregnancy are more likely than women with no psychiatric illness to have a probable depressive and/or anxiety disorder during pregnancy and the postpartum period, and to have greater worries over gestational weight gain [8–11]. Compared to healthy controls, having BN or BED confers an increased risk of induced abortions and miscarriages, respectively, and the latter eating disorder subtype may also increase the risk of having higher birth weight and large for gestational age infants [12,13]. Maternal AN has been found to be associated with an increased risk of miscarriage, low birth weight infants, suspect fetal distress and perinatal death [3,14–16]; however it is still not clear whether this eating disorder may also increase the risk of prematurity [12,15,16].

Few clinical trials have tested pharmacotherapy options for treatment of patients with eating disorders. Although there is no evidence supporting general use of antidepressants or antipsychotics for the treatment of AN, selective serotonin reuptake inhibitor (SSRI) antidepressants seem to moderately reduce the symptoms of BN and BED, but exert little effect on full recovery [17–21]. Previous research in clinical settings has shown that 13% and 49% of women with AN use antipsychotics an antidepressants, respectively [22]. Nevertheless, little is known about the extent of use of psychotropics in a population-based setting.

The use of medication in women with eating disorders has as far as we know not been explored in relation to pregnancy. Inadequate evidence-based counseling about medication safety in pregnancy and negative information framing may led women to discontinue needed medication once pregnant [23]. However, since pharmacotherapy with psychotropics might reduce pregnancy-related exacerbation of eating disorder symptoms such as dieting or vomiting, their effect would probably be beneficial for both mother and fetus rather than detrimental. Since extreme dieting, compensatory behaviors, or psychiatric comorbidity among patients with eating disorders are associated with several painful conditions, including gastrointestinal complaints [24,25], a comprehensive understanding of medication use beyond psychotropics including analgesics and gastrointestinal medication in women with eating disorders, is essential to ensure maternal-fetal health.

Thus, this study investigated patterns of use of psychotropic, analgesic, and gastrointestinal medications before, during, and after pregnancy across eating disorder subtypes, and explored the relationship between eating disorders and use of these specific medications during pregnancy and the postpartum, including whether there was a direct association between eating disorders and medication use or whether the association was indirect, e.g. via an underlying maternal depression and anxiety. We hypothesized a higher extent of medication use in the pregnancy and postpartum periods among women with eating disorders compared to healthy controls.

## Materials and Methods

### Study population and data collection

This study is based on the Norwegian Mother and Child Cohort Study (MoBa) and on records in the Medical Birth Registry of Norway (MBRN). MoBa is a prospective population-based pregnancy cohort study conducted by the Norwegian Institute of Public Health [26].

Participants were recruited from all over Norway from 1999–2008. The women consented to participation in 40.6% of the pregnancies [27]. The cohort now includes 114,500 children, 95,200 mothers and 75,200 fathers. Participants were recruited through a postal invitation in connection with a routine ultrasound examination offered to all pregnant women in Norway at 17–18 weeks of gestation. The current study is based on version 7 of the quality-assured data files released for research including women who delivered between 1999 and 2009. Informed consent was obtained from each participant. The study was approved by The Regional Committee for Medical Research Ethics and the Norwegian Data Inspectorate.

**Fig 1:**
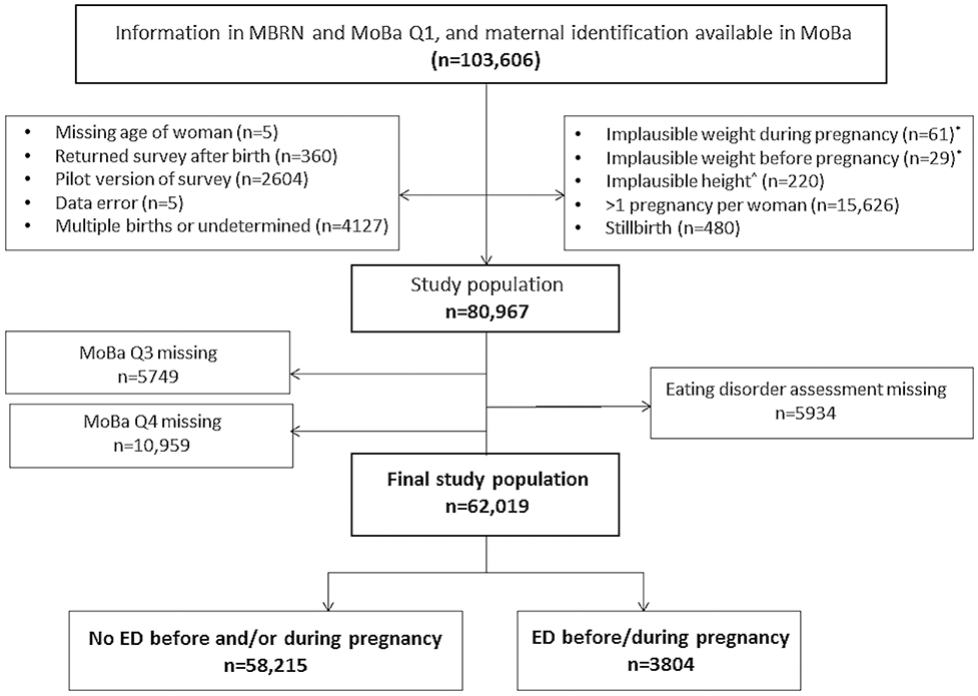
Flow-chart to achieve final study population. Conditions may overlap: excluded participants are not mutually exclusive. *Weight either < 30 Kg or > 300 Kg; ^^^Height < 100 cm.

The MBRN is based on compulsory notification of all live births, stillbirths and induced abortions and includes information on pregnancy, delivery and neonatal health [28]. Data from MoBa was linked to the MBRN via the women’s personal identification number. The analysis population for this study included women who had a record in MBRN, and had answered three self-administered MoBa questionnaires [29]. The first (Q1) and third (Q3) questionnaires were completed in gestational weeks 13–17 and 30, respectively; the fourth questionnaire (Q4), concerning the period from gestational week 30 and onwards, was distributed when the infant was six months old [26,29]. Among those who agreed to participate in the MoBa, the response rate was 95% for Q1, 92% for Q3, and 87% for Q4 [26]. The exclusion criteria and flow-chart to achieve the final population analysis are outlined in Fig 1.

### Measures

#### Eating disorder

Q1 included items on eating disorders and disordered eating behaviors designed in accordance with the DSM-IV criteria [30]. These items have been utilized in several publications based upon on MoBa data [12,31–36]. The same diagnostic questions have also been used in previous studies on eating disorders in the Norwegian Twin Panel [37,38] and they yielded prevalence estimates and comorbidity profiles similar to those seen in another population-based sample [39]. In our analysis population, respondents completed Q1 at a median of 17.1 weeks of gestation (interquartile range 16.0–18.6 weeks). Diagnostic algorithms and hierarchies were constructed to define the presence of eating disorders in the six months prior to pregnancy (retrospective assessment) and during pregnancy. Broadly defined AN was defined as meeting the DSM-IV criteria for AN with the exception of amenorrhea and also having a body mass index (BMI) <18.5 at the time of low weight. Our definition of AN is more in accordance with DSM-5 since the amenorrhea criterion is eliminated. It was not possible to classify AN during pregnancy because of the missing BMI criterion due to pregnancy-induced weight gain. The other eating disorder categories included: broadly defined BN, endorsing at least a weekly frequency of binge eating and either purging (vomiting, laxatives) or non-purging (exercise, fasting) compensatory behaviors; broadly defined BED, at least a weekly frequency of binge eating in the absence of compensatory behaviors; and eating disorder not otherwise specified-purging subtype (EDNOS-P), purging at least weekly in the absence of binge eating. Questions for binge eating included both eating an unusually large amount of food and the feeling of loss of control. The frequency criteria for binge eating and purging in BN, BED, and EDNOS-P differed from the DSM-IV criteria but reflect the new DSM-5 criteria (once a week instead of twice a week). As the symptom profile for many women changed in the interval before pregnancy and during pregnancy, the following diagnostic hierarchy was applied in order to assign only one diagnosis to each woman: AN, BN, EDNOS-P, BED, and no eating disorder. All individuals who met AN criteria before pregnancy were categorized as AN regardless of presentation during pregnancy. Those who met BN criteria either before or during pregnancy and who did not meet AN criteria prior to pregnancy were categorized as BN. If not classified as AN or BN, those who met criteria for EDNOS-P before or during pregnancy and did not endorse binge eating at either time were categorized as EDNOS-P. Similarly, individuals who endorsed BED and did not endorse purging during or before pregnancy were included in the BED group. Group assignment was made only when all responses were available to ensure accurate classification.

### Outcome assessment

Self-reported information about type and timing of medication use was available from the MoBa Q1, Q3 and Q4 [29]. Respondents were asked to report medication use for numerous chronic, short-term, and pregnancy-related conditions as free entry text, along with the timing of use (six months before pregnancy; first, second and third trimesters; and two time periods postpartum [0–3 and 4–6 months after childbirth]). All medications recorded in Q1, Q3 and Q4 were grouped according to the Anatomical Therapeutic Chemical (ATC)[40] codes, as outlined in S1 Table, into: psychotropics (i.e., antidepressants, antipsychotics, anxiolytics and sedatives), gastrointestinal medications (i.e., antacids, drugs for peptic ulcer and gastro-esophageal reflux disease, laxatives), and analgesics (i.e., opioids, acetaminophen and other antipyretics, and nonsteroidal anti-inflammatory drugs [NSAIDs]). When multiple drugs were used and multiple timings checked, we considered the drugs to be used in all time periods. Our outcome measures (dichotomous ‘Yes/No’) were: a) medication use at any time “during pregnancy”, and “postpartum” separately, irrespective of the respondents’s medication use status in the other time periods; b) incident use of medications “during pregnancy only” (i.e., women who started taking the medication in pregnancy and were not using that medication neither before nor after pregnancy) and “postpartum only” (i.e., women who started taking the medication postpartum and were not using that medication neither before nor during pregnancy).

### Assessment of maternal mental health

Symptoms of depression and anxiety during pregnancy and postpartum were measured via the short versions of The Hopkins Symptom Checklist-25 (SCL-25): the Symptom Checklist-5 (SCL- 5) in Q1, and the Symptom Checklist-8 (SCL-8) in Q3 and Q4 [41,42]. The scale is considered a reliable screening instrument for depression and anxiety as defined by the ICD-10 [43]. Both SCL-5 and SCL-8 are highly correlated to the SCL-25 [42,44]. For each item of the scales, a score from 1 to 4 can be assigned. Whenever the respondent completed more than a half of the items, imputed values were generated on both instruments via utilization of the estimation-maximization algorithm. Values were imputed for 1.4%, 5.4%, and 8.9% of the study population in SCL-5 (Q1), SCL-8 (Q3), and SCL-8 (Q4), respectively. For all three instruments, the mean score was separately computed. Presence of depressive and anxiety symptoms during pregnancy was defined by a score greater than 2.0 in the SCL-5 and greater than 1.85 in the SCL-8 [41]. The mean scores for the SCL-5 in Q1 and the SCL-8 in Q3 were summed (mean sum score) in order to measure symptoms of depression and anxiety throughout the pregnancy.

### Assessment of potential confounders and mediators

Maternal socio-demographics (i.e., age, educational level, socio-economic status, BMI at conception, weight gain during pregnancy, weight decrease after childbirth, illnesses during pregnancy), life-style characteristics (i.e., smoking status until gestational week 30 and alcohol intake during early pregnancy) and the degree of maternal depressive and anxiety symptoms during pregnancy (mean sum score of SCL-5 and SCL-8) and postpartum (mean score of the SCL-8) were all analyzed as potential confounders or mediators. Confounding and mediating factors were identified with the aid of directed acyclic graphs (DAGs) using DAGitty version 2.2 (one DAG for each medication-outcome pair) [45]. Our assumptions were: eating disorder status before and/or during pregnancy precedes maternal symptoms of depression and anxiety during pregnancy; eating disorder status before and/or during pregnancy determines BMI at conception. These assumptions applied to all the eating disorder subtypes.

### Statistical analysis

All statistical analyses were performed by using the Statistical Package for the Social Sciences (SPSS) version 22.0 (IBM SPSS Statistics). The Pearson chi-square or Fisher exact test, and the Student's t-test were utilized to compare proportions and mean scores between independent groups, respectively. Because of the numerous analyses, we undertook a conservative approach and considered p-values of ≤ 0.01statistically significant.

The Generalized Estimating Equations (GEE) with a Poisson distribution [46] was used to test differences in medication use across the eating disorder subtypes. In the first set of analyses we explored medication use “during pregnancy” and “postpartum” separately. In the second set, we assessed incident use of medications “during pregnancy only” and “postpartum only”. In the two sets of analyses we carried out the following steps: we first computed crude relative risks (RR) with 99% CI. Then, we entered in Model 1 the minimal sufficient adjustment set of variables (i.e., age, socioeconomic, status and educational level for all medication groups) for estimating the total association between eating disorders and the outcomes of interest. In a sensitivity analysis we included BMI at conception as additional covariate in Model 1 (because of the uncertainty in the direction of the association between BMI and eating disorders); however, the observed results did not differ substantially from the main analyses. In Model 2 we entered the set of confounders from Model 1 plus additional covariates (e.g., maternal depressive and anxiety symptoms, BMI, weight gain in pregnancy, alcohol use during early pregnancy and smoking until gestational week 30) in order to estimate the direct association between eating disorders and the outcomes of interest. Data are presented as crude and adjusted RR if there were at least three cases of women with eating disorders exposed to the specific medication groups.

## Results

### Population characteristics

A total of 62,019 women were included in this study (Fig 1). Those excluded from the analysis because of missing eating disorder assessment (n = 5,934, 9.6%) were significantly older, had less education, lower socio-economic status, and higher BMI at conception than those included. Women completing Q1 but not Q3 (i.e., they were lost to follow-up at gestational week 30) were more likely than women completing both Q1 and Q3 to have symptoms of depression and anxiety around gestational week 17 (11.0% vs. 6.9%, respectively; p<0.001). Similarly, the prevalence of eating disorders was significantly higher (p<0.001) among women who were lost to follow-up than individuals remaining in the study (AN: 0.2% vs. 0.1%), BN (1.5% vs. 1.0%), EDNOS-P (0.3% vs. 0.1%), and BED (6.4% vs. 5.1%). The analysis of attrition bias in relation to women lost to follow-up at six months postpartum showed that women completing Q1 and Q3 but not Q4 had a significantly higher burden of depressive symptoms around gestational week 30 compared to women who did complete Q4 (10.7% vs. 6.7%, respectively; p<0.001).

In our study population, the prevalence of eating disorder subtypes before and/or during pregnancy was: 0.09% AN (n = 54), 0.94% BN (n = 585), 0.10% EDNOS-P (n = 61) and 5.00% BED (n = 3,104). The remaining 93.87% did not present with any eating disorders (reference group). Maternal socio-demographics, life-style factors, morbidities, and mental health characteristics across the eating disorder subtypes are outlined in Table 1. Women within the AN, BN, EDNOS-P, and BED groups more frequently had less education and lower socio-economic status than the reference group, and showed significantly higher rates of depressive and anxiety symptoms throughout the pregnancy (Table 1).

**Table 1:** Maternal sociodemographics, morbidities and mental health across the eating disorder subtype (n = 62,019)^†^.

	AN (n = 54)	BN (n = 585)	EDNOS-P (n = 61)	BED (n = 3,104)	No ED (n = 58,215)
**Sociodemographics and life-style factors**					
**Age (in years)** *(Mean ± sd)*	26.8 ± 4.7*	29.5 ± 4.7^‡^	28.0 ± 5.3*	30.1 ± 4.7	30.0 ± 4.5
**BMI at conception** ^a^ *(Mean ± sd)*	18.2 ± 0.6*	24.1 ± 4.3	23.7 ± 4.5	25.9 ± 5.1*	23.9 ± 4.1
**Previous children (%)**					
No	37 (68.5)	324 (55.4)	39 (63.9)	1,517 (48.9)*	32,226 (55.4)
Yes	17 (31.5)	261 (44.6)	22 (36.1)	1,587 (51.1)	25,989 (44.6)
**Marital status (%)**					
Married/cohabiting	52 (96.3)	540 (93.6)*	51 (85.0)*	2,958 (95.8)*	56,139 (96.9)
Others	2 (3.7)	37 (6.4)	9 (15.0)	130 (4.2)	1,822 (3.1)
**Educational level** ^**b**^ **(%)**					
Primary/secondary school	28 (57.1)*	244 (44.9)*	30 (50.0)^‡^	1,292 (43.8)*	18,514 (33.5)
University/higher degree	21 (42.9)	299(55.1)	30 (50.0)	1,657 (56.2)	36,691 (66.5)
**Minimum household income (%)**					
0–499,999 NOK ($0–77,999)	24 (46.2)*	114 (21.3)^‡^	17 (32.1)^‡^	632 (21.9)*	9,976 (18.3)
500–999,999 NOK ($78,000–155,999)	26 (50.0)	382 (71.4)	33 (62.3)	2,113 (73.1)	40,986 (75.3)
>1 million NOK ($156,000)	2 (3.8)	27 (5.0)	1 (1.9)	102 (3.5)	2,900 (5.3)
Unknown	-	12 (2.2)	2 (3.8)	45 (1.6)	567 (1.0)
**Smoking during pregnancy** ^**c**^ **(%)**					
No	42 (77.8)	457 (78.1)	49 (80.3)	2,456 (79.1)	45,567 (78.3)
Yes	3 (5.6)	47 (8.0)	6 (9.8)	249 (8.0)	4,710 (8.1)
Missing	9 (16.7)	81 (13.8)	6 (9.8)	399 (12.9)	7,938 (13.6)
**Alcohol use during pregnancy** ^**d**^ **(%)**					
No	37 (68.5)	433 (74.0)	49 (80.3)	2,184 (70.4)	41,171 (70.7)
Yes	10 (18.5)	65 (11.1)	5 (8.2)	341 (11.1)	6,340 (10.9)
Missing	7 (13.0)	87 (14.9)	7 (11.5)	579 (18.7)	10,704 (18.4)
**Illnesses during pregnancy (%)**					
Pelvic girdle/back/shoulder/other pains	45 (83.3)	483 (82.6)*	49 (80.3)	2,516 (81.1)*	42,937 (73.8)
Headache/migraine	25 (46.3)	261 (44.6)*	24 (39.3)	1,298 (41.8)*	19,937 (34.2)
Gastrointestinal disorders^**e**^	41 (75.9)	406 (69.4)*	43 (70.5)	2,159 (69.6)*	35,803 (61.5)
**Mental health during pregnancy (%)**					
Depressive and anxiety symptoms^f^	12 (22.2)*	99 (16.9)*	8 (13.1)*	222 (7.2)*	1,255 (2.2)

Abbreviations: AN (anorexia nervosa), BN (bulimia nervosa), EDNOS-P (eating disorder not otherwise specified, purging type), BED (binge-eating disorder), ED (eating disorder); BMI (body mass index); NOK (Norwegian Kroners).

^a^The BMI is the weight in kilograms divided by the square of the height in meters: underweight: <18.5 kg/m^2^; normal weight: 18.5–24.9 kg/m^2^; overweight: 25.0–29.9 kg/m^2^; obese≥30 kg/m^2^.

^b^Primary or secondary education: <10 years (primary) or 10–12 years (secondary) of education; University degree or higher: college or university education.

^c^Indicates smoking until gestational week 30.

^d^Indicates alcohol use at the beginning of pregnancy, until gestational week 17.

^e^Includes heartburn/reflux, duodenal/stomach ulcers, Crohn disease/ulcerative colitis and other gastrointestinal problems.

^f^Indicates scoring over the cut-off point at both gestational weeks 17 (5-items Hopkins symptoms checklist SCL-5≥2) and 30 (8-items Hopkins symptoms checklist SCL8≥1.85).

^†^Numbers may not add up to total because of missing values (<5%). For the variables “smoking” and “alcohol use during pregnancy”, missing values were up to 18% and a “missing category” was therefore created. The group “medication users with no ED” is the referent group for all analyses.

*Indicates p-value ≤ 0.001

^‡^Indicates p-value ≤ 0.01.

### Patterns of medication use

**Fig 2:**
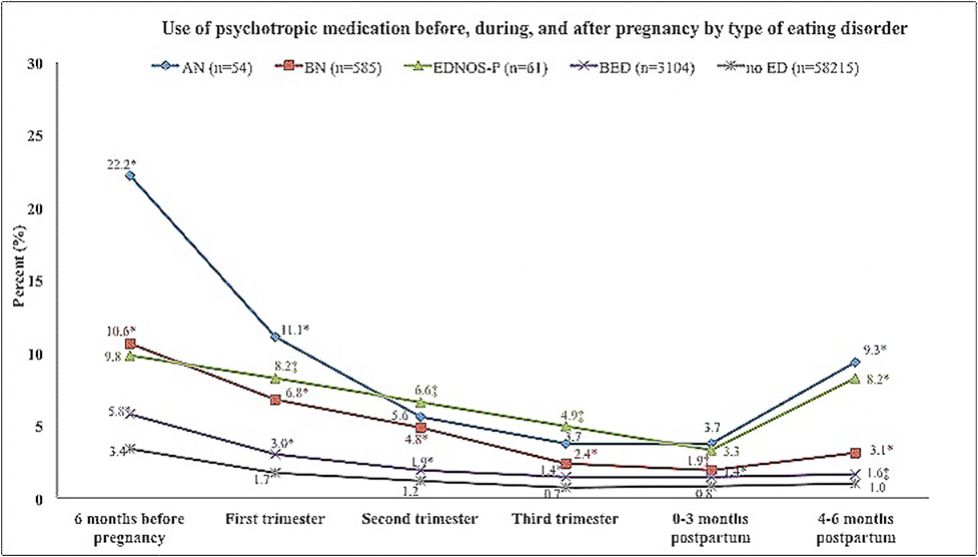
Use of psychotropic medications before, during, and after pregnancy by type of eating disorder^†^. Abbreviations: AN (anorexia nervosa), BN (bulimia nervosa), EDNOS-P (eating disorder not otherwise specified, purging type), BED (binge-eating disorder), ED (eating disorder). ^†^Psychotropic medications include antidepressants, antipsychotics, anxiolytics and hypnotics and sedatives. *Indicates p-value ≤0.001; ^‡^Indicates p-value ≤0.01.

Fig 2 outlines the extent of psychotropic medication use overtime across the various eating disorder subtypes. Women with AN or EDNOS-P reported the highest rate of psychotropic medication use prior, during and after pregnancy. Use of psychotropics decreased during pregnancy across all eating disorders compared to the period before conception; at 4–6 months postpartum the AN and EDNOS-P groups were characterized by a significant increase in such use (mainly anxiolytics and sedatives) (Fig 2 and S2 Table). The extent of use of the individual psychotropic medications overtime, including regular use at all time periods and across the various eating disorder subtypes is outlined in S2 Table. Overall, antidepressants comprised the medication class most widely used before, during, and after pregnancy.

S1 and S2 Figs outline the extent of use of gastrointestinal drugs and analgesics, respectively, according to timing and across the eating disorder subtypes. Patterns of use for the individual subgroups within gastrointestinal drugs and analgesics are shown in S3 and S4 Tables, respectively. Women with any eating disorder were characterized by a high use of gastrointestinal drugs during pregnancy (especially in the second and third trimester) and postpartum. Compared to the reference group, all eating disorder subtypes were characterized by a higher rate of laxative use at some point before, during, or after pregnancy (S3 Table).

Even though not always significantly different, use of analgesics was at almost all time points higher among women with AN than the reference counterpart (S2 Fig). Women with BED were characterized by a significantly higher use of any type of analgesics before, as well as during and after pregnancy. Also, women with AN, BN or BED were more likely than the reference group to use acetaminophen and other antipyretics at all time periods (S4 Table).

### Association between eating disorders and medication use in pregnancy

Table 2 outlines the measure of association between the eating disorder subtypes and use of specific medication groups during pregnancy. After adjusting for confounding factors (Model 1), women with AN, BN, EDNOS-P, and BED had a significant 5.6-, 4.0-, 3.6- and 1.7-fold increased likelihood, respectively, to use psychotropics during pregnancy compared to the reference group. Having BN was directly associated with use of psychotropics during pregnancy (1.8-fold magnitude) compared to not having any eating disorder. In a sub-analysis by psychotropic subgroup, BED was found to be significantly directly associated with use of antidepressants during pregnancy (aRR: 1.45, 99% CI: 1.01–2.08), while BN had such effect on use of anxiolytics and sedatives (aRR: 2.36, 99% CI: 1.26–4.41) compared to women with no eating disorder. Only BN was significantly directly associated with incident use of psychotropics during pregnancy (Model 2, aRR: 2.25, 99% CI: 1.17–4.32) compared to the reference group.

**Table 2:** Measure of association between eating disorder subtypes and medication use during pregnancy^†^.

		Model 1*	Model 2^‡^
Medication group	Crude RR (99% CI)	Adjusted RR (99% CI)	Adjusted RR (99% CI)
***Psychotropics***			
AN (n = 8)	**6.08 (2.62–14.12)**	**5.63 (2.30–13.76)**	1.98 (0.74–5.26)
BN (n = 61)	**4.28 (3.11–5.89)**	**4.01 (2.84–5.66)**	**1.81 (1.21–2.71)**
EDNOS-P (n = 7)	**4.71 (1.88–11.80)**	**3.63 (1.21–10.85)**	2.77 (0.95–8.08)
BED (n = 135)	**1.78 (1.42–2.24)**	**1.71 (1.34–2.17)**	1.09 (0.82–1.44)
No ED (n = 1,419)	Referent	Referent	Referent
***Gastrointestinal drugs***			
AN (n = 14)	1.13 (0.63–2.05)	1.30 (0.73–2.33)	1.52 (0.83–2.81)
BN (n = 167)	**1.25 (1.05–1.48)**	**1.27 (1.06–1.52)**	1.05 (0.84–1.30)
EDNOS-P (n = 20)	1.43 (0.89–2.30)	1.52 (0.93–2.49)	**1.68 (1.09–2.59)**
BED (n = 869)	**1.23 (1.13–1.32)**	**1.19 (1.10–1.29)**	1.01 (0.92–1.11)
No ED (n = 13,306)	Referent	Referent	Referent
***Analgesics***			
AN (n = 33)	1.24 (0.94–1.64)	1.42 (1.08–1.87)	1.27 (0.92–1.74)
BN (n = 338)	**1.17 (1.07–1.28)**	**1.19 (1.08–1.31)**	1.09 (0.98–1.21)
EDNOS-P (n = 35)	1.16 (0.88–1.55)	1.09 (0.78–1.53)	1.08 (0.75–1.57)
BED (n = 1,760)	**1.15 (1.10–1.20)**	**1.11 (1.06–1.16)**	1.03 (0.98–1.09)
No ED (n = 28,733)	Referent	Referent	Referent

^†^Indicates medication use at any point during pregnancy, irrespective of the medication use status before or after pregnancy. The RR is computed when there are at least 3 women exposed to the medication group of interest. Statistically significant results are in bold.

*Model 1: Adjustment done for maternal age (as continuous variable), socioeconomic status and educational level (for all medication groups).

^‡^Model 2: Adjustment done for all covariates in Model 1 with addition of alcohol use during pregnancy, smoking during pregnancy, weight gain during entire pregnancy (as continuous variable), BMI at conception (as continuous variable), depressive and anxiety symptoms throughout the pregnancy (as continuous variable), pain ailments in pregnancy (for analgesics), and gastrointestinal disorders during pregnancy (for gastrointestinal drugs).

Women with BN or BED presented a significant 1.3- and 1.2-fold increased likelihood, respectively, for taking gastrointestinal drugs during pregnancy compared to the reference group (Model 1). However, only the EDNOS-P subtype was significantly directly associated with this outcome (specifically for antacids and laxatives).

Compared to the reference group, having BN or BED were significantly associated with a modest increased likelihood to use analgesics during pregnancy (Model 1, 11–19% increased risk); however, none of the eating disorder subtypes was directly associated with this outcome (Model 2). In the second set of analysis, women with BED presented a small significant likelihood to be incident users of analgesics during pregnancy (Model 1, aRR: 1.14, 99% CI: 1.02–1.28) compared to women with no eating disorder, although the association was not direct.

### Association between eating disorders and medication use postpartum

Table 3 outlines the measure of association between the eating disorder subtypes and use of specific medication groups postpartum. Women with AN, BN, EDNOS-P, and BED presented a significant 9.5-, 2.4-, 7.2- and 1.5-fold increased likelihood, respectively, to use psychotropics in the period 0–6 months after delivery compared to the reference group (Model 1). Only the EDNOS-P subtype was directly associated with this outcome (Model 2, 4.5-fold magnitude). In the sub-analysis on type of psychotropics, AN and EDNOS-P were directly associated with an increased likelihood of using anxiolytics/sedatives postpartum (Model 2, aRR: 5.11, 99% CI: 1.53–17.01; aRR: 6.77, 99% CI: 1.41–32.53, respectively) compared to women with no eating disorders.

**Table 3:** Association between eating disorder subtypes and use of medication in the postpartum period^†^.

		Model 1*	Model 2^‡^
Medication group	Crude RR (99% CI)	Adjusted RR (99% CI)	Adjusted RR (99% CI)
***Psychotropics***			
AN (n = 7)	**10.00 (4.02–24.94)**	**9.55 (3.58–25.48)**	2.87 (0.91–9.08)
BN (n = 21)	**2.77 (1.58–4.85)**	**2.44 (1.29–4.61)**	0.93 (0.42–2.04)
EDNOS_P (n = 5)	**6.33 (2.09–19.16)**	**7.16 (2.41–21.32)**	**4.47 (1.18–16.86)**
BED (n = 62)	**1.54 (1.10–2.16)**	**1.46 (1.02–2.10)**	0.87 (0.56–1.35)
No ED (n = 754)	Referent	Referent	Referent
***Gastrointestinal drugs***			
AN (n = 5)	2.14 (0.71–6.42)	2.43 (0.83–7.18)	1.84 (0.43–7.78)
BN (n = 44)	**1.74 (1.19–2.53)**	**1.82 (1.22–2.72)**	**1.63 (1.03–2.58)**
EDNOS_P (n = 1)	-	-	-
BED (n = 155)	1.16 (0.94–1.42)	1.19 (0.95–1.48)	1.17 (0.92–1.49)
No ED (n = 2,520)	Referent	Referent	Referent
***Analgesics***			
AN (n = 24)	1.19 (0.81–1.76)	1.20 (0.79–1.84)	1.16 (0.71–1.90)
BN (n = 237)	1.09 (0.95–1.24)	1.12 (0.98–1.29)	0.98 (0.84–1.15)
EDNOS_P (n = 20)	0.88 (0.55–1.41)	0.89 (0.53–1.49)	0.77 (0.40–1.48)
BED (n = 1,284)	**1.11 (1.05–1.17)**	**1.12 (1.06–1.19)**	1.06 (0.99–1.13)
No ED (n = 21,710)	Referent	Referent	Referent

^†^Indicates medication use at any point in the period 0–6 months after delivery, irrespective of the medication use status before or during pregnancy. The RR is computed when there are at least 3 women exposed to the medication group of interest. Statistically significant results are in bold.

*Model 1: Adjustment done for maternal age (as continuous variable), socioeconomic status and educational level (for all medication groups).

^‡^Model 2: Adjustment done for all covariates in Model 1 with addition of weight decrease six months after delivery and breastfeeding status, depressive and anxiety symptoms postpartum, and cesarean section (for analgesics).

In Model 1, BN was significantly associated with a 1.8-fold increased likelihood to take gastrointestinal drugs postpartum compared to the reference group, and also showed a direct association with this outcome (Model 2, 1.6-fold magnitude). Women with BED, even though in a modest magnitude, were more likely than the reference group to use analgesics postpartum (1.2-fold increased risk); however, the association was not direct. No eating disorder was significantly associated with incident use of gastrointestinal drugs or analgesics postpartum.

## Discussion

To our knowledge this is the first population-based study addressing the extent of medication use among women with eating disorders in the period before, during, and after pregnancy. Several of our findings are important for clinical practice. First, knowledge that use of psychotropic medication, especially antidepressants, was common among women with any eating disorder in the preconception period as well as during pregnancy and postpartum may assist clinicians when following-up or counseling female patients with eating disorders. Indeed, women with eating disorders, either pregnant or planning a pregnancy, might be in special need of evidence-based counseling about the benefit-risk ratio of gestational exposure to antidepressants or other psychotropics, and that of untreated psychiatric illness. To date very little is known about the distinct effects of treated versus untreated eating disorders on perinatal outcomes [15,16]; however the detrimental impact of untreated maternal depression, which is highly comorbid with eating disorders, on maternal-fetal health has been documented [47,48].

Second, women with AN or EDNOS-P presented the highest rate of psychotropic drug use at all time periods investigated, which may be due to a high degree of psychiatric comorbidity compared to the other groups of women. Women with AN were also those with the highest extent of regular use (i.e., before, during and after pregnancy) of psychotropics (5.6%), which is not completely unexpected since more than one out of five women with AN presented symptoms of depression and anxiety during pregnancy. Kaye et al.[49] showed in a double-blind placebo-controlled trial that use of fluoxetine may be useful in improving outcome and preventing relapse of patients with AN after weight restoration; since most women with AN are weight restored during the course of the pregnancy, SSRI antidepressants, and in particular fluoxetine, may actually be more beneficial in this setting than before conception.

Third, women with EDNOS-P or AN had a 6.8- and 5.1-fold increased likelihood to use pharmacotherapy with sedatives/anxiolytics in the postpartum period, even after cancelling out the effect of factors such as weight decrease postpartum or depressive and anxiety symptoms. The substantial physical changes accompanying motherhood may represent a special challenge for women with AN, being characterized by a profound fear of gaining weight and by a distorted perception of body shape. Although about 50% of women with AN or EDNOS-P have been shown to remit at 18 months postpartum [35], little is known about the course of these disorders in the earlier postpartum period. Women with AN or EDNOS-P were found to lose the gestational weight more quickly than controls over the first six months postpartum [36], thus for these women a return to restrictive weight control behaviors and a worsening of the anxiety symptomatology in the early postpartum period, requiring use of sedatives/anxiolytics, cannot be excluded.

Fourth, women with BED were characterized by an extensive use of analgesics before, during and after pregnancy. In the multivariate analysis, though, BED was not directly associated with analgesic use during pregnancy or postpartum, suggesting that other factors, namely depressive and anxiety symptoms, pain ailments, BMI, weight change during pregnancy and postpartum, rather than the binging behavior, might constitute the driving factors for using analgesics.

Lastly, our study revealed that use of laxatives is high among women with any eating disorders not only before pregnancy, but also during pregnancy and the postpartum, raising concerns about the impact of this practice on their own health and that of their unborn children.

Our observed rates of use of psychotropics in the preconception period were lower than those found in three previous studies among women with AN (53%), BED (18%), or all eating disorders (96.7%)[22,50,51]; different recruitment strategies, that is, population-based recruitment in the present study versus clinical research recruitment in others, country-specific therapeutic traditions and access to special care in different countries, could probably explain these discrepancies. Factors such as pregnancy planning might have also deflated our estimates; because of fear to harm the unborn child and elevated risk perception of medication exposure, many women may discontinue their needed pharmacotherapy during pregnancy or when attempting to conceive [52,53].

The lack of drug utilization studies among women with eating disorders during pregnancy unfortunately precludes any comparison of our observed estimates of use of psychotropics with the existing literature. Yet, our crude estimates of use of antidepressants during pregnancy among women with AN or EDNOS-P (AN: 13.0%, EDNOS-P: 8.2%) were not substantially different from those expected to occur among women with symptoms of depression in Norway (about 9–13%)[54–56], although use was lower among women with BN or BED (BN: 5.6%, BED: 2.8%). Indeed, the rates of depressive and anxiety symptomatology throughout pregnancy were significantly higher among women with any eating disorder than in the reference group, ranging from 22.2% for AN to 7.2% for BED.

Although the association among eating disorders, alcohol consumption, and smoking is well-established [28], this was not reflected in our pregnant sample. In fact, the crude estimates of alcohol consumption during early pregnancy and smoking until the end of pregnancy among women with eating disorders were not significantly different from those of the reference group, although alcohol use was higher in the AN group. We also tested whether eating disorders were directly associated with use of psychotropics, i.e. after adjusting for factors such as maternal underlying depressive and anxiety symptomatology, weight gain and life-style factors, including alcohol consumption during pregnancy. Then, only BN was directly associated with use and incident use of psychotropics during pregnancy (1.8- and 2.3-fold, respectively) compared to controls. Since antidepressants have shown some effects in reducing the binge-eating and vomiting behaviors and fluoxetine is the only medication approved for treatment of BN [57], this finding is expected. On the other hand, incident use of psychotropics might also represent a proxy of increased severity of a pre-existing or an incident case of BN. A previous study [31] using the same data source found that the most common pattern for BN was remission or partial remission of symptoms from the pre-pregnancy period to early pregnancy, and incident cases were rare. Given this scenario, we cannot exclude the possibility that pharmacotherapy with psychotropics might have contributed, at least to some extent, to remission of symptoms among women with BN. Also, women with BN might have sought specialist care and treatment once pregnant for the well-being of the fetus. Two previous studies have for example shown that use of dietary supplements and nutritional intake during pregnancy were similar among women with and without eating disorders [32,58], underscoring how these women do their utmost to ensure the well-being of the developing fetus.

The extent of use of gastrointestinal medication observed in our study was high across all the eating disorders; this finding may reflect a higher burden of gastrointestinal bothers during pregnancy among women with eating disorders than in the healthy counterpart, but it raises several concerns. In particular, women with BED were more often users of gastrointestinal medications during pregnancy (antacids, laxatives, and drugs for gastroesophageal reflux disease [GERD]) and postpartum (drugs for GERD), though not prior to pregnancy, suggesting a possible augmentation in severity or frequency of bingeing episodes during these periods, or more intense pregnancy-related bothers in the gastrointestinal tract secondary to the binge. Prior research using the MoBa cohort [31] has in fact shown that most women with BED experienced continuation of symptoms rather than remission during pregnancy compared to the period before conception, and incident cases were not uncommon. In our multivariate model, though, no direct associations between BED and use of gastrointestinal medications during pregnancy and postpartum were found, implying the importance of indirect factors, namely depressive and anxiety symptoms, weight gain or decrease, BMI and gastrointestinal concerns, on these associations. EDNOS-P, on the other hand, was directly associated with use of gastrointestinal medications during pregnancy (mostly antacids), which may be secondary to regurgitation episodes or to an intensification of purging behavior (i.e., vomiting) during pregnancy or, as shown by Torgersen et al., to the higher odds for these women to experience pregnancy-related vomiting [59].

In line with prior research showing an association between moderate to severe pain and eating disorders [60], we found that use of analgesics before, during and after pregnancy was high across all eating disorder subtypes. However, the multivariate analysis showed that when accounting for factors such as depressive and anxiety symptoms, pain disorders and weight increase or decrease, none of the eating disorders were directly associated with any analgesic use neither during nor after pregnancy. The higher extent of use of NSAIDs in the third trimester among women with AN, BN or EDNOS-P, however, deserves attention. Women should be advised against use of NSAIDs in the third trimester since use of NSAIDs after week 32 has been associated with premature closure of the ductus arteriosus, oligohydramnios, and inhibition of labor [61].

Frequent follow-ups and support with treatment by a multidisciplinary team including obstetricians, psychiatrists, and psychotherapists is of critical importance for women with eating disorders, especially in a vulnerable phase of life such as pregnancy and motherhood. The high burden of psychiatric comorbidity and the extensive medication use among these women deserves attention: clinicians are encouraged to query female patients about their medication-taking behavior and provide evidence-based counseling about the risk of medication exposure versus the risk of untreated psychiatric illness during pregnancy and postpartum. Sub-optimal treatment of maternal psychiatric illness might lead to adverse outcomes such as a relapse of the disorder, poor life-style or inadequate compliance with prenatal care, which are all harmful factors for both mother and child. In moderate to severe cases of psychiatric illness pharmacotherapy may be necessary [62].

### Strengths and Limitations of the Study

The MoBa study encompasses several strengths and limitations. Data collection was carried out prospectively, avoiding the risk of recall bias. Use of medications in the period from gestational week 30 to childbirth was the only information collected retrospectively (in Q4), and may therefore suffer of recall bias. However, the impact of misclassification of use of SSRIs (the most common psychotropics in our sample) in late pregnancy on risk estimates was assessed as minimal [63]. The collection of a vast array of health-related and sociodemographic information enabled us to take into account several potential confounders and mediators. The utilization of DAGs permitted a proper selection of confounding factors for the multivariate models, thus diminishing the risk of over-adjustment. Symptoms of depression and anxiety were measured at two time points in pregnancy and at six months postpartum via utilization of validated instruments, i.e. the SCL-5 and SCL-8, which are reliable screening tools [41–44].

On the other hand, our study has several limitations that should be considered when interpreting the results. Assessment of broadly defined eating disorders was based on women´s self-report, however the questions posed to the study participants were consistent with diagnostic criteria [30]. Other psychometric instruments (e.g., the SCOFF questionnaire) could have been used to identify individuals with eating disorders; however, the eating disorder hierarchy employed in our study has been widely used [31,32,34–36]. Categorization based on diagnostic interviews may have yielded better diagnostic information, although this approach was not feasible in the context of the MoBa study given the large sample size. However, women may deny the presence of stigmatized behaviors such as purging in face-to-face interviews [64]. It is plausible that women with eating disorders who participated in MoBa may represent the healthier end of the eating disorder severity spectrum because they had to be well enough to conceive and participate overtime. Our prevalence estimate of AN was somewhat lower than the point estimate reported in another study in pregnancy (0.1% vs. 0.5%, respectively). Such discrepancy could be ascribed, at least in part, to the different diagnostic criteria utilized. In the present study, the mean BMI at conception among women with AN may be an indicator of mild anorexic symptomatology; indeed, women with severe anorexia are most likely not well enough to conceive and participate in a population-based research study. MoBa is a population-based study, and therefore the BMI among women with broadly defined AN is expected to be higher than the BMI expected in a clinical sample of patients with AN. Nonetheless, the gradient of severity of eating disorders in participants included in clinical versus population-based studies is not unexpected given the well-documented differences in severity and comorbidity across population- and clinic-based investigations [65]. The present study also suffers from attrition bias, losing participants with more severe eating disorders and/or depressive and anxiety symptomatology during the follow-up period. The MoBa study has a low response rate (40.6% of all women invited), with a possible self-selection of the healthiest women to the study. On the other hand, among those who accepted the invitation, the response rate is high [26]. A previous study [27] has thoroughly examined self-selection and its potential for bias by comparing the MoBa study population with the total Norwegian birthing population, and concluded that although the prevalence estimates could not necessarily be generalized, the measures of associations tested were valid in the MoBa study. We cannot, however, rule out that some of the association found here could be influenced by selection bias. Women excluded from the analysis because of missing items for the eating disorder assessment had a more unfavorable profile than the included counterpart, implying a plausible exclusion of women with more severe eating disorder symptoms. Our sample was small for the AN and EDNOS-P groups, limiting the statistical power of most analyses. Information on medication dosage is not available in the MoBa study and data about duration of exposure is not always adequate. Information about type and timing of medication use is self-reported, thus dependent on the accuracy of the women’s reporting. However, the validity of self-reported use of antidepressants in the MoBa study has been found to be reliable [63]. Information about ongoing psychological or psychotherapeutic treatment for the various eating disorders was also not available in the MoBa study. Symptoms of depression and anxiety were measured by two self-assessment instruments; although such measurements cannot replace a clinical interview and are not designed to measure perinatal mood specifically, they provide a reliable measure of the severity of these psychiatric conditions [41,43]. Lastly, we cannot rule out the presence of unmeasured factors confounding the association between eating disorders and medication use, and therefore cannot conclude with regard to whether the associations found reflect causal relationships.

In view of our results, women with particularly low or high BMI should be queried about their eating and medication-taking behaviors during the routine prenatal care check-ups. Pregnancy represents an important window for recognition of psychiatric symptoms, including eating disorders. During this phase of life, women with eating disorders may be motivated for treatment for the well-being of the unborn child, and this could also prevent the recurrence of symptoms in the post-partum period. Further studies are needed to explore whether women with eating disorders self-medicate or receive prescriptions for gastrointestinal and analgesic medications during pregnancy, as well as their medication-taking behavior in the later postpartum period. Future studies should also evaluate the distinct effect of medicated and not medicated eating disorders on immediate perinatal and long-term neurodevelopmental outcomes and should focus on how obstetricians, psychiatrists, pharmacists, and midwives can form multidisciplinary teams to ensure that women with eating disorders in pregnancy receive the care and support they need for themselves and their children during this important phase of life.

## Conclusions

Our study indicated that psychotropics, especially antidepressants, are widely used by women with eating disorders in the period before, during, and after pregnancy. In particular, women with AN or EDNOS-P were those most often taking psychotropics, which could partly be related to the high psychiatric comorbidity. Women with BN were more likely than healthy controls to initiate pharmacotherapy with psychotropics during pregnancy, even after accounting for the effect of indirect factors. Similarly, AN or EDNOS-P were directly associated with use of anxiolytics/sedatives over the six month period after childbirth. While women with BED were characterized by an extensive use of analgesics before, during and after pregnancy, use of laxatives was high among women with any eating disorder at all time periods investigated.

## Supporting Information

S1 Fig Use of gastrointestinal medications before, during, and after pregnancy by type of eating disorder^†^. Abbreviations: AN (anorexia nervosa), BN (bulimia nervosa), EDNOS-P (eating disorder not otherwise specified, purging type), BED (binge-eating disorder), ED (eating disorder). †Gastrointestinal medications include antacids, drugs for peptic ulcer and gastroesophageal reflux disease, and laxatives. *Indicates p-value ≤0.001; ‡Indicates p-value ≤0.01.
(TIF)

S2 Fig Use of analgesic medications before, during, and after pregnancy by type of eating disorder^†^. Abbreviations: AN (anorexia nervosa), BN (bulimia nervosa), EDNOS-P (eating disorder not otherwise specified, purging type), BED (binge-eating disorder), ED (eating disorder). †Analgesics comprise centrally acting analgesics (i.e. opioids and antipyretics) and NSAIDs. *Indicates p-value ≤0.001; ‡Indicates p-value ≤0.01.
(TIF)

S1 Table Categorization of the medications groups included in the analyses according to the ATC classification system. Abbreviations: ATC: Anatomical Therapeutic Chemical; GERD: Gastroesophageal reflux disease.
(PDF)

S2 Table Use of psychotropic medication subgroups before, during, and after pregnancy by type of eating disorder^†^. Abbreviations: AN (anorexia nervosa), BN (bulimia nervosa), EDNOS-P (eating disorder not otherwise specified, purging type), BED (binge-eating disorder), ED (eating disorder). ^†^The “No eating disorder” group is the reference group for all analyses. *Indicates p-value ≤0.001; ^‡^Indicates p-value ≤0.01.
(PDF)

S3 Table Use of gastrointestinal medication subgroups before, during, and after pregnancy by type of eating disorder^†^. Abbreviations: AN (anorexia nervosa), BN (bulimia nervosa), EDNOS-P (eating disorder not otherwise specified, purging type), BED (binge-eating disorder), ED (eating disorder); GERD: Gastroesophageal reflux disease. Drugs for GERD include H2-receptor antagonists, prostaglandins, proton pump inhibitors, and other drugs for GERD (i.e., sucralfate and alginic acid). ^†^The “No eating disorder” group is the reference group for all analyses. *Indicates p-value ≤0.001; ^‡^Indicates p-value ≤0.01.
(PDF)

S4 Table Use of any analgesic subgroups before, during, and after pregnancy by type of eating disorder^†^. Abbreviations: AN (anorexia nervosa), BN (bulimia nervosa), EDNOS-P (eating disorder not otherwise specified, purging type), BED (binge-eating disorder), ED (eating disorder), NSAIDs (nonsteroidal anti-inflammatory drugs). Antipyretics include acetylsalicylic acid, acetaminophen alone or as a combination product. ^†^The “No eating disorder” group is the reference group for all analyses. *Indicates p-value ≤0.001; ^‡^Indicates p-value ≤0.01.
(PDF)
