# Supplementary figures and images for: Medication Use before, during, and after Pregnancy among Women with Eating Disorders: A Study from the Norwegian Mother and Child Cohort Study
Source: PLoS One. 2015 Jul 22;10(7):e0133045. doi: 10.1371/journal.pone.0133045 (PMC4511584; doi:10.1371/journal.pone.0133045)

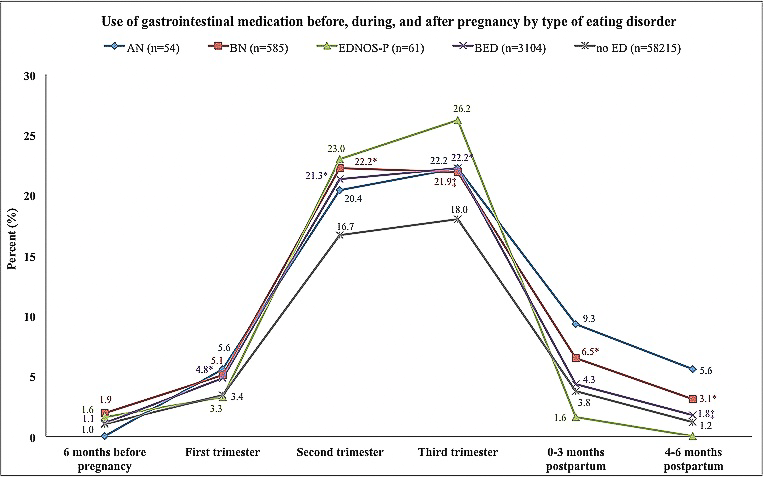

Supplement: S1 Fig — Abbreviations: AN (anorexia nervosa), BN (bulimia nervosa), EDNOS-P (eating disorder not otherwise specified, purging type), BED (binge-eating disorder), ED (eating disorder). †Gastrointestinal medications include antacids, drugs for peptic ulcer and gastroesophageal reflux disease, and laxatives. *Indicates p-value ≤0.001; ‡Indicates p-value ≤0.01. (TIF) [file pone.0133045.s001.tif]

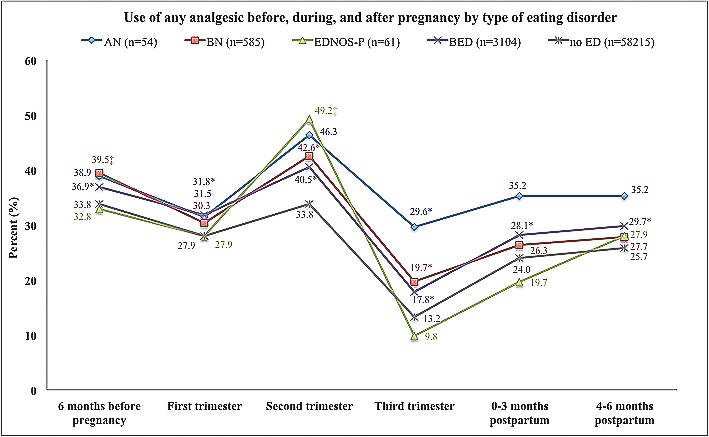

Supplement: S2 Fig — Abbreviations: AN (anorexia nervosa), BN (bulimia nervosa), EDNOS-P (eating disorder not otherwise specified, purging type), BED (binge-eating disorder), ED (eating disorder). †Analgesics comprise centrally acting analgesics (i.e. opioids and antipyretics) and NSAIDs. *Indicates p-value ≤0.001; ‡Indicates p-value ≤0.01. (TIF) [file pone.0133045.s002.tif]
