# Supplementary material for: Medication Use before, during, and after Pregnancy among Women with Eating Disorders: A Study from the Norwegian Mother and Child Cohort Study
Source: PLoS One. 2015 Jul 22;10(7):e0133045. doi: 10.1371/journal.pone.0133045 (PMC4511584; doi:10.1371/journal.pone.0133045)
Supplement: S1 Table — Abbreviations: ATC: Anatomical Therapeutic Chemical; GERD: Gastroesophageal reflux disease. (PDF) [file pone.0133045.s003.pdf]

Medication group	ATC code
**Psychotropics**	
Antidepressants	N06AA, N06AB, N06AX
Antipsychotics	N05A
Anxiolytics and sedatives	N05BA, N05BB, N05BE, N05CF, N05CD
**Gastrointestinal drugs**	
Antacids	A02AC, A02AD, A02AH
Drugs for peptic ulcer and GERD	A02BA, A02BB, A02BC, A02BX
Laxatives	A06AA, A06AB, A06AC, A06AD, A06AG, A06AH, A06AX
**Analgesics**	
Opioids	N02A
Acetaminophen and other antipyretics	N02B
Antiinflammatory and antirheumatic products, non-steroids (NSAIDs)	M01AB, M01AC, M01AE, M01AG, M01AH, M01AX
