# Supplementary material for: Medication Use before, during, and after Pregnancy among Women with Eating Disorders: A Study from the Norwegian Mother and Child Cohort Study
Source: PLoS One. 2015 Jul 22;10(7):e0133045. doi: 10.1371/journal.pone.0133045 (PMC4511584; doi:10.1371/journal.pone.0133045)
Supplement: S2 Table — Abbreviations: AN (anorexia nervosa), BN (bulimia nervosa), EDNOS-P (eating disorder not otherwise specified, purging type), BED (binge-eating disorder), ED (eating disorder). †The “No eating disorder” group is the reference group for all analyses. *Indicates p-value ≤0.001; ‡Indicates p-value ≤0.01. (PDF) [file pone.0133045.s004.pdf]

Psychotropic medication group	AN (n=54)	BN (n=585)	EDNOS-P (n=61)	BED (n=3104)	No ED (n=58215)
	n (%)	*n (%)*	*n (%)*	*n (%)*	*n (%)*
**Antidepressants**					
Before pregnancy	**9 (16.7)***	**46 (7.9)***	**6 (9.8)‡**	**156 (5.0)***	1474 (2.5)
First trimester	**5 (9.3)***	**24 (4.1)***	**5 (8.2)***	**66 (2.1)***	469 (0.8)
Second trimester	**3 (5.6)‡**	**15 (2.6)***	**3 (4.9)‡**	**33 (1.1)***	254 (0.4)
Third trimester	1 (1.9)	**9 (1.5)***	2 (3.3)	**28 (0.9)***	185 (0.3)
Any time during pregnancy	**7 (13.0)***	**33 (5.6)***	**5 (8.2)***	**86 (2.8)***	586 (1.0)
0-3 months postpartum	-	**9 (1.5)***	1 (1.6)	**25 (0.8)***	236 (0.4)
4-6 months postpartum	2 (3.7)	**13 (2.2)***	2 (3.3)	**35 (1.1)***	359 (0.6)
Before, during and after pregnancy	1 (1.9)	**7 (1.2)***	1 (1.6)	**17 (0.5)‡**	159 (0.3)
**Anxiolytics and sedatives**					
Before pregnancy	**5 (9.3)***	**21 (3.6)***	1 (1.6)	34 (1.1)	572 (1.0)
First trimester	1 (1.9)	**11 (1.9)***	-	16 (0.5)	257 (0.4)
Second trimester	-	**8 (1.4)‡**	1 (1.6)	17 (0.5)	207 (0.4)
Third trimester	-	**6 (1.0)‡**	1 (1.6)	**19 (0.6)***	160 (0.3)
Any time during pregnancy	1 (1.9)	**23 (3.9)***	2 (3.3)	**46 (1.5)***	494 (0.8)
0-3 months postpartum	1 (1.9)	2 (0.3)	1 (1.6)	20 (0.6)	218 (0.4)
4-6 months postpartum	**3 (5.6)‡**	7 (1.2)	**3 (4.9)‡**	22 (0.7)	257 (0.4)
Before, during and after pregnancy	1 (1.9)	-	**-**	3 (0.1)	44 (0.1)
**Antipsychotics**					
Before pregnancy	1 (1.9)	**5 (0.9)‡**	1 (1.6)	9 (0.3)	137 (0.2)
First trimester	1 (1.9)	**9 (1.5)‡**	-	19 (0.6)	344 (0.6)
Second trimester	-	7 (1.2)	-	13 (0.4)	294 (0.5)

Third trimester	1 (1.9)	1 (0.2)	-	4 (0.1)	79 (0.1)
Any time during pregnancy	2 (3.7)	**12 (2.1)**‡	-	24 (0.8)	481 (0.8)
0-3 months postpartum	1 (1.9)	1 (0.2)	-	4 (0.1)	51 (0.1)
4-6 months postpartum	1 (1.9)	2 (0.3)	-	3 (0.1)	60 (0.1)
Before, during and after pregnancy	-	1 (0.2)	-	-	18 (0.03)
