# Supplementary material for: Medication Use before, during, and after Pregnancy among Women with Eating Disorders: A Study from the Norwegian Mother and Child Cohort Study
Source: PLoS One. 2015 Jul 22;10(7):e0133045. doi: 10.1371/journal.pone.0133045 (PMC4511584; doi:10.1371/journal.pone.0133045)
Supplement: S3 Table — Abbreviations: AN (anorexia nervosa), BN (bulimia nervosa), EDNOS-P (eating disorder not otherwise specified, purging type), BED (binge-eating disorder), ED (eating disorder); GERD: Gastroesophageal reflux disease. Drugs for GERD include H2-receptor antagonists, prostaglandins, proton pump inhibitors, and other drugs for GERD (i.e., sucralfate and alginic acid). †The “No eating disorder” group is the reference group for all analyses. *Indicates p-value ≤0.001; ‡Indicates p-value ≤0.01. (PDF) [file pone.0133045.s005.pdf]

Gastrointestinal medication group	AN (n=54)	BN (n=585)	EDNOS-P (n=61)	BED (n=3104)	No ED (n=58215)
	n (%)	*n (%)*	*n (%)*	*n (%)*	*n (%)*
**Antacids**					
Before pregnancy	-	2 (0.3)	-	5 (0.2)	81 (0.1)
First trimester	-	7 (1.2)	1 (1.6)	40 (1.3)	551 (0.9)
Second trimester	2 (3.7)	37 (6.3)	7 (11.5)	**266 (8.6)***	3536 (6.1)
Third trimester	6 (11.1)	43 (7.4)	9 (14.8)	**284 (9.1)***	3923 (6.7)
Any time during pregnancy	6 (11.1)	49 (8.4)	10 (16.4)	**348 (11.2)***	4805 (8.3)
0-3 months postpartum	-	**5 (0.9)‡**	-	14 (0.5)	135 (0.2)
4-6 months postpartum	-	**6 (1.0)‡**	-	11 (0.4)	124 (0.2)
Before, during and after pregnancy	-	**-**	-	-	6 (0.01)
**Drugs for peptic ulcer and GERD**					
Before pregnancy	-	5 (0.9)	-	29 (0.9)	453 (0.8)
First trimester	1 (1.9)	7 (1.2)	1 (1.6)	**57 (1.8)***	654 (1.1)
Second trimester	6 (11.1)	67 (11.5)	6 (9.8)	**344 (11.1)***	5258 (9.0)
Third trimester	6 (11.1)	74 (12.6)	6 (9.8)	**394 (12.7)***	6315 (10.8)
Any time during pregnancy	7 (13.0)	92 (15.7)	8 (13.1)	**473 (15.2)***	7503 (12.9)
0-3 months postpartum	-	2 (0.3)	-	**19 (0.6)‡**	184 (0.3)
4-6 months postpartum	-	-	-	**19 (0.6)‡**	173 (0.3)
Before, during and after pregnancy	-	-	-	2 (0.1)	27 (0.05)
**Laxatives**					
Before pregnancy	-	**5 (0.9)***	1 (1.6)	1 (0.03)	76 (0.1)
First trimester	2 (3.7)	**21 (3.6)***	-	67 (2.2)	936 (1.6)
Second trimester	5 (9.3)	**42 (7.2)***	3 (4.9)	**154 (5.0)‡**	2247 (3.9)

Third trimester	4 (7.4)	**35 (6.0)***	5 (8.2)	107 (3.4)	1738 (3.0)
Any time during pregnancy	6 (11.1)	**61 (10.4)***	6 (9.8)	**215 (6.9)‡**	3279 (5.6)
0-3 months postpartum	5 (9.3)	**31 (5.3)‡**	1 (1.6)	112 (3.6)	1945 (3.3)
4-6 months postpartum	**3 (5.6)‡**	**12 (2.1)***	-	30 (1.0)	406 (0.7)
Before, during and after pregnancy	**-**	**-**	-	-	12 (0.02)
