# Supplementary material for: Medication Use before, during, and after Pregnancy among Women with Eating Disorders: A Study from the Norwegian Mother and Child Cohort Study
Source: PLoS One. 2015 Jul 22;10(7):e0133045. doi: 10.1371/journal.pone.0133045 (PMC4511584; doi:10.1371/journal.pone.0133045)
Supplement: S4 Table — Abbreviations: AN (anorexia nervosa), BN (bulimia nervosa), EDNOS-P (eating disorder not otherwise specified, purging type), BED (binge-eating disorder), ED (eating disorder), NSAIDs (nonsteroidal anti-inflammatory drugs). Antipyretics include acetylsalicylic acid, acetaminophen alone or as a combination product. †The “No eating disorder” group is the reference group for all analyses. *Indicates p-value ≤0.001; ‡Indicates p-value ≤0.01. (PDF) [file pone.0133045.s006.pdf]

Analgesic medication group	AN (n=54)	BN (n=585)	EDNOS-P (n=61)	BED (n=3104)	No ED (n=58215)
	n (%)	*n (%)*	*n (%)*	*n (%)*	*n (%)*
**Opioids**					
Before pregnancy	3 (5.6)	**20 (3.4)**‡	1 (1.6)	**90 (2.9)***	1065 (1.8)
First trimester	2 (3.7)	10 (1.7)	1 (1.6)	**45 (1.4)***	505 (0.9)
Second trimester	3 (5.6)	**12 (2.1)**‡	3 (4.9)	**48 (1.5)**‡	592 (1.0)
Third trimester	1 (1.9)	6 (1.0)	-	**32 (1.0)**‡	378 (0.6)
Any time during pregnancy	4 (7.4)	19 (3.2)	3 (4.9)	**100 (3.2)***	1215 (2.1)
0-3 months postpartum	1 (1.9)	9 (1.5)	1 (1.6)	52 (1.7)	697 (1.2)
4-6 months postpartum	1 (1.9)	9 (1.5)	1 (1.6)	**64 (2.1)***	644 (1.1)
Before, during and after pregnancy	1 (1.9)	1 (0.2)	-	**14 (0.5)***	108 (0.2)
**Acetaminophen and other antipyretics**					
Before pregnancy	20 (37.0)	190 (32.5)	19 (31.1)	**952 (30.7)**‡	16555 (28.4)
First trimester	16 (29.6)	167 (28.5)	15 (24.6)	**906 (29.2)***	14998 (25.8)
Second trimester	25 (46.3)	**241 (41.2)***	**29 (47.5)**‡	**1210 (39.0)***	19040 (32.7)
Third trimester	**16 (29.6)***	**112 (19.1)***	6 (9.8)	**533 (17.2)***	7352 (12.6)
Any time during pregnancy	32 (59.3)	**326 (55.7)***	34 (55.7)	**1676 (54.0)***	27500 (47.2)
0-3 months postpartum	18 (33.3)	144 (24.6)	10 (16.4)	**813 (26.2)***	12859 (22.1)
4-6 months postpartum	18 (33.3)	148 (25.3)	13 (21.3)	**829 (26.7)***	13480 (23.2)
Before, during and after pregnancy	**12 (22.2)**‡	**84 (14.4)**‡	5 (8.2)	**425 (13.7)***	6322 (10.9)
**NSAIDs**					
Before pregnancy	9 (16.7)	86 (14.7)	7 (11.5)	**417 (13.4)**‡	6799 (11.7)
First trimester	4 (7.4)	29 (5.0)	2 (3.3)	**166 (5.3)**‡	2489 (4.3)
Second trimester	1 (1.9)	22 (3.8)	4 (6.6)	**121 (3.9)***	1559 (91.3)
Third trimester	2 (3.7)	5 (0.9)	1 (1.6)	**59 (1.9)***	554 (1.0)

**Analgesic medication group**	**AN (n=54)**	**BN (n=585)**	**EDNOS-P (n=61)**	**BED (n=3104)**	**No ED (n=58215)**
	n (%)	*n (%)*	*n (%)*	*n (%)*	*n (%)*
Any time during pregnancy	5 (9.3)	53 (9.1)	4 (6.6)	**296 (9.5)***	4057 (7.0)
0-3 months postpartum	3 (5.6)	34 (5.8)	3 (4.9)	**179 (5.8)**‡	2767 (4.8)
4-6 months postpartum	5 (9.3)	44 (7.5)	5 (8.2)	**213 (6.9)***	3108 (5.3)
Before, during and after pregnancy	1 (1.9)	12 (2.1)	-	37 (1.2)	639 (1.1)
